# The TBI-AD/ADRD Caregiver Support Intervention (TACSI): Protocol of a Pilot Randomized Controlled Trial Evaluation of a Remote Intervention for Family Caregivers

**DOI:** 10.2196/81125

**Published:** 2026-03-17

**Authors:** Samantha Ostenso, Robyn W Birkeland, Elizabeth Albers, Katie W Louwagie, Allison Iwan, Sherry S Chesak, Joseph E Gaugler

**Affiliations:** 1 Families and Long Term Care, School of Public Health, University of Minnesota, 420 Delaware St SE, D351 Mayo, Minneapolis, MN, 55455, United States, 1 9522618286; 2 School of Nursing, University of Minnesota, Minneapolis, MN, United States

**Keywords:** traumatic brain injury, dementia, caregiver, mixed methods, support

## Abstract

**Background:**

Across the United States, there are millions of informal (ie, unpaid) caregivers helping individuals with Alzheimer Disease/Alzheimer Disease and Related Dementias (AD/ADRD), traumatic brain injury (TBI), or both. TBI is a risk factor for developing, and often co-occurs with, AD/ADRD. In the next decade, more informal caregivers will have to navigate the complexities of the dual diagnosis of TBI and AD/ADRD. Currently, there is a paucity of interventions for caregivers dealing with this dual diagnosis. Our team designed the TBI-AD/ADRD caregiver support intervention (TACSI) as a support program to meet the needs of those providing care to individuals with dual diagnoses of TBI and AD/ADRD.

**Objective:**

This pilot randomized controlled trial (RCT) evaluates the preliminary efficacy and successful implementation of TACSI, a 6-session semistructured psychosocial and psychoeducational program, over a 6-month period.

**Methods:**

This pilot RCT uses an embedded mixed methods randomized controlled evaluation. Caregivers of individuals with a dual diagnosis of AD/ADRD and a history of TBI are randomly assigned to the TACSI (n=41) or a usual care control condition (n=39). Primary outcomes include perceived burden, caregiver relationship satisfaction, and caregiving mastery. Secondary outcomes include caregiver self-efficacy, well-being, and personal resources. Qualitative elements (up to 20 semistructured interviews at the conclusion of the 6-month evaluation and an open-response item at follow-up surveys) allow for better interpretation of quantitative results and understanding of TACSI’s mechanisms of benefit.

**Results:**

Recruitment for the study was completed in October 2024. Survey collection and intervention were completed in June 2025. Data analysis will follow.

**Conclusions:**

Having a dual diagnosis of AD/ADRD and a history of TBI is relatively common. However, there are currently no caregiver support interventions specific to this dual diagnosis. It is important to support these caregivers as they encounter the unique challenges of managing TBI and AD/ADRD. This pilot RCT will help determine if the TACSI program can help to increase caregiving skills and strategies and provide support to improve mood and reduce stress for caregivers of individuals with AD/ADRD and a history of TBI, and also determine if it is feasible to proceed to a full-scale RCT.

## Introduction

In the United States in 2025, an estimated 7.2 million adults 65 years and older have an Alzheimer Disease/Alzheimer Disease and Related Dementias (AD/ADRD) diagnosis, while each year approximately 2.8 million Americans are diagnosed with a traumatic brain injury (TBI) [1,2]. Each of these conditions can significantly impact one’s well-being and ability to complete activities of daily living (ADL) and function independently. Informal (unpaid) caregivers, typically family, often step forward to provide support to these individuals. Over 11 million adults in the United States provide informal care for someone with AD/ADRD [1]. Additionally, about 2.5 million adults serve as informal caregivers for someone who has experienced a TBI [3].

Research has found that individuals with a history of TBI are more likely to develop AD/ADRD and other neurological disorders than those without TBI [4-7]. In fact, a cohort study following a proportion of the general population for 5 years found that participants with TBI had 1.68 times the risk of developing dementia compared with those without TBI [8]. Given the increased risk of developing AD/ADRD from TBI, it is likely that many caregivers provide support to an individual with a dual diagnosis of AD/ADRD and a history of TBI.

On their own, the care demands associated with AD/ADRD and TBI have negative mental, social, financial, and physical health impacts on caregivers as evidenced in decades of research [9-16]. Together, these conditions can be even more complex and challenging, creating new and unique needs for the caregiver and care recipient. For example, individuals with post-TBI dementia have an increased risk of co-occurring mental health conditions such as mixed affective disorders, bipolar disorder, and post-traumatic stress disorder [17]. They may also experience more agitation, irritability, disinhibition, and depressive symptoms than people with AD/ADRD alone [17]. Individuals with post-TBI dementia also experience more falls and mobility issues than those solely diagnosed with dementia [17] and often require more medications, medical visits, and hospital days than individuals with solely TBI [17]. Unsurprisingly, given these additional stressors, informal caregivers for veterans with the dual diagnoses of TBI and AD/ADRD reported experiencing symptoms of anxiety and depression and felt severely burdened by their duties [17].

Clearly, these unique dual-diagnosis caregivers need support. There are many evidence-based interventions designed to support dementia caregivers, including the Resources for Enhancing Alzheimer’s Caregiver Health (REACH) intervention, a structured multicomponent program tailored to caregiver needs that has been shown to increase the quality of life of ethnically diverse dementia caregivers [18,19]. Other multicomponent and psychoeducational interventions for dementia caregivers have also been found to reduce caregiver depressive symptoms and sense of burden and improve their well-being, confidence, and social support [20]. Fewer interventions exist and have been evaluated for their efficacy for TBI caregivers [21-24]. The limited research on these interventions for TBI caregivers suggests that the inclusion of education, skill-building, social support, coping strategies, and resilience holds potential for increasing quality of life and caregiver mastery [22,23,25-27].

Despite these interventions for these individual diagnoses, there is a complete lack of support interventions designed specifically for caregivers of individuals with a dual diagnosis of AD/ADRD and TBI. A 2020 randomized controlled trial (RCT) protocol paper described an intervention at the primary care level for Veteran caregiver dyads with a care recipient who had either TBI or dementia, but not both [28]. To our knowledge, there is only one other intervention aimed at supporting TBI-AD/ADRD dual diagnosis caregivers, and it is also being developed. Researchers in the Veterans Administration are currently evaluating this intervention, REACH Hope (a modification of the REACH program), for AD/ADRD and TBI dual diagnosis caregivers.

We designed the TBI-AD/ADRD caregiver support intervention (TACSI) as an intervention to meet the unique needs of those providing care to individuals with dual diagnoses of a history of TBI and AD/ADRD. TACSI is a psychoeducational and psychosocial support intervention designed to help informal caregivers build caregiving skills and strategies while providing education and support. TACSI is a semistructured program, tailored to meet the needs of individual caregivers as well as the family. The intervention offers one-on-one coaching designed to increase caregiver understanding of TBI-AD/ADRD; provides strategies to improve engagement and communication with the care recipient, family, and health care team; and develops skills to better manage caregiver stress and increase external support.

A 3-month preliminary pilot study established the feasibility, acceptability, and appropriateness of TACSI among 15 caregivers of individuals with TBI and AD/ADRD [29]. Following minor revisions to the program based on the preliminary pilot, the purpose of this study is to deploy a pilot RCT design with 80 caregivers to evaluate the preliminary efficacy and successful implementation of TACSI over a 6-month period.

## Methods

### Overview

Coinvestigators from Mayo Clinic and the Minneapolis Veterans Affairs Health Care System (MVAHCS) collaborated with the University of Minnesota (UMN) team in designing the TACSI protocol.

The UMN maintains oversight of all study procedures, data collection, data management, and dissemination. In addition to ongoing review of protocol modifications, the UMN Institutional Review Board requires an annual continuing review process, with approval contingent upon review and approval of a study submission regarding participant accrual, withdrawal, and other study-related issues. An independent safety monitor provided additional study oversight, reviewing the annual data and safety monitoring report. Quarterly reports, including enrollment and safety updates, were submitted to the Department of Defense. This protocol was reported following the SPIRIT (Standard Protocol Items: Recommendations for Interventional Trials) reporting guidelines (Checklist 1) [30].

### Ethical Considerations

The UMN Institutional Review Board approved the study protocol for the UMN and Mayo Clinic (STUDY00014886); current version 7, June 4, 2025. The MVAHCS obtained site-specific approval (1641329). All study procedures (consent, data collection, and intervention delivery) were conducted remotely with participating caregivers residing in the United States. Based on the caregiver’s preference, consent was completed either electronically via the secure survey platform, Qualtrics, via telephone, or mailed with telephone consent.

All electronic data (eg, administrative tracking logs, survey data, and coaches’ notes) from potential and enrolled participants were maintained in secure environments (ie, Qualtrics, secure project folder, and Box). Participant data and their identifying information were kept in separate files. Links to identifiable data will be destroyed 2‐3 years after data collection is complete. All data on the team’s computers was encrypted and protected by strong passwords only accessible to the UMN research team. The team used Duo 2-step verification to access VPN/Secure servers. Any hard copy forms were stored in a file cabinet in an office that was locked at all times. Interview audio recordings were deleted from secure recording devices after they were uploaded to Box and a secure server. Transcripts were created by a HIPAA (Health Insurance Portability and Accountability Act)-compliant organization and deidentified.

Upon completion of the study, caregivers were compensated for their participation. Caregivers received US $25 for each completed survey (T0, T1, and T2), for a total of up to US $75. An additional US $25 was provided to those caregivers in the treatment group who completed a final semistructured interview. Separate compensation for completion of the treatment receipt checklist (TRC) was not provided.

### Aims

Prior to initiating this pilot study of TACSI, the study team developed and implemented TACSI for 15 family members of persons with a dual diagnosis of TBI & AD/ADRD to assess the feasibility, acceptability, and appropriateness of TACSI over a 3-month period (phase I). The results of the pilot study supported the feasibility, acceptability, and appropriateness of TACSI [29].

In this study (phase II), the specific aims were to use a pilot RCT design with 80 caregivers to evaluate the implementation and preliminary efficacy of a refined version of TACSI over a 6-month period. Specifically, we sought to evaluate the impact of TACSI on primary subjective stressors, including caregiver perceived burden, relationship satisfaction, ideology, and mastery (primary outcomes), primary objective stressors (assistance with ADL/instrumental activities of daily living), and well-being, resource usage, and coping strategies.

### Design

**Figure 1.**
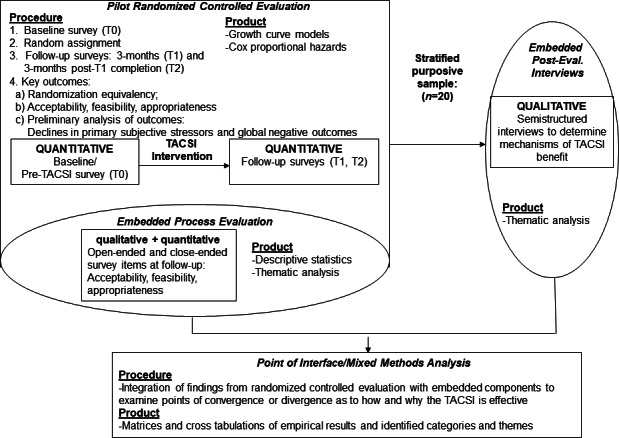
Mixed methods design. TACSI: TBI (traumatic brain injury)-AD/ADRD (Alzheimer disease and related dementias) caregiver support intervention.

This embedded experimental mixed methods study used a pilot RCT design to determine the feasibility and performance of TACSI with 80 caregivers of persons with TBI-ADRD. The mixed methods approach integrated elements of quantitative and qualitative data collection at multiple time points throughout the study for a more robust analysis, enriching our understanding of the caregiver experience in comparison to solely collecting qualitative or quantitative data (Figure 1) [31,32].

Inclusion of qualitative data provides pertinent context for interpretation of quantitative results and rich insight into the intervention’s mechanisms of benefit [33,34]. The mixed methods pilot RCT design allowed for the evaluation of appropriateness and sufficiency of the recruitment, screening, randomization, treatment receipt, and retention processes (all appropriate pilot RCT goals over a 6-month follow-up period) [35].

### Setting

Study survey administration was offered via web or mail to be completed in hard copy. Two trained coaches from the UMN delivered TACSI to caregivers in the treatment group via secure video conferencing (ie, Zoom) or telephone. Finally, caregivers selected to participate in a semistructured interview could complete the interview by videoconference or telephone.

### Participant Eligibility

Eligible caregivers were those who (1) cared for someone with a dual diagnosis of progressive cognitive decline (eg, AD/ADRD) and history of a TBI, (2) provided the most help to the care recipient (or shared the role equally), (3) were willing to participate in the TACSI evaluation, (4) spoke English, (5) were 21 y or older, (6) were not currently participating in other 1:1 psychosocial consultation specific to caregiving, and (7) resided in the United States. Caregivers were excluded if they endorsed a new or worsening mental health condition for which they were not receiving ongoing treatment. If using a psychotropic medication, caregivers needed to have been on a stable dose for at least 3 months prior to study enrollment.

### Processes, Interventions, and Procedures

#### Recruitment

Based on the target population of those caring for patients with a dual diagnosis of TBI and AD/ADRD, the MVAHCS and Mayo Clinic generated internal data requests for patient records using *ICD* (*International Classification of Diseases*) codes. Specific *ICD-10* (*International Statistical Classification of Diseases, Tenth Revision*) codes included G30, G30.9–Alz Disease, F03-Unspecified dementia, S06.2–Diffuse brain injury, and S06.3–Focal brain injury. Potential participants were listed as caregivers in the patient’s medical records.

**Figure 2.**
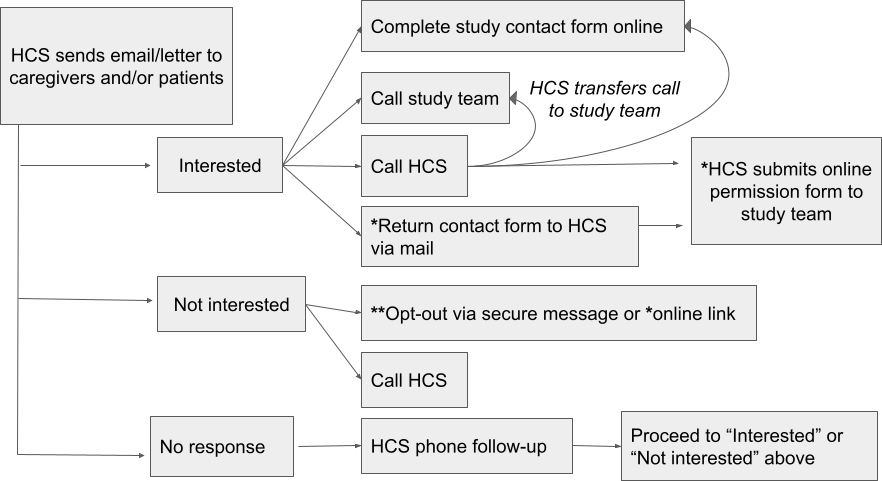
HCS recruitment diagram. HCS: health care system. *Applies to Mayo Sites Only; ** Applies to VA (Veterans Affairs) site only.

The 2 health care systems (HCSs) were responsible for making initial contact to describe the study. The UMN study team met weekly with staff from Mayo Clinic and MVAHCS throughout study recruitment. Specifics of the HCS contact protocol slightly varied by site. Figure 2 shows the general flow of contacts at both sites. Patients and caregivers affiliated with Mayo Clinic received initial contacts via email, mail, or both, and follow-up telephone calls from the HCS for those contacts who did not respond. Contacts could opt out of study-related communication (via telephone or web-based form) or indicate study interest by completing a web-based study contact form, calling the UMN team study telephone or HCS, or returning a mailed permission form to the HCS using a pre-paid envelope. For those providing permission, Mayo Clinic shared contact information with the UMN study team.

Patients and caregivers affiliated with the MVAHCS received contacts similarly. The MVAHCS required a letter or encrypted email to be sent prior to study-related follow-up telephone calls by the HCS. Contacts could opt out of further study-related communication. Since the MVAHCS could not share contact information for interested caregivers directly with the UMN study team, the MVAHCS protocol required interested contacts to alert the study team directly via a web-based form or a call (by the caregiver or by transferring their call to the study line). To facilitate recruitment, staff at either HCS could provide a phone transfer to the UMN study telephone number for interested contacts to discuss screening with the research team.

The majority of participants were recruited through the MVAHCS or Mayo Clinic. In addition to HCS recruitment solely based on diagnosis codes, caregivers of someone with a dual diagnosis of TBI and AD/ADRD could express interest in participating in this research study when recruited by MVAHCS or through study postings on ClinicalTrials.gov and StudyFinder. Interested caregivers could call or email the study team directly or complete a web-based permission to contact form to obtain more information about the study.

Each potential participant completed a telephone-based screening procedure to determine eligibility. Only caregivers who were not referred from the MVAHCS or Mayo Clinic were required to answer eligibility questions related to their care recipient’s diagnoses to ensure the presence of a dual diagnosis of TBI-AD/ADRD. If eligible, the staff member described the study activities and proceeded to the consent procedures.

#### Enrollment

**Figure 3.**
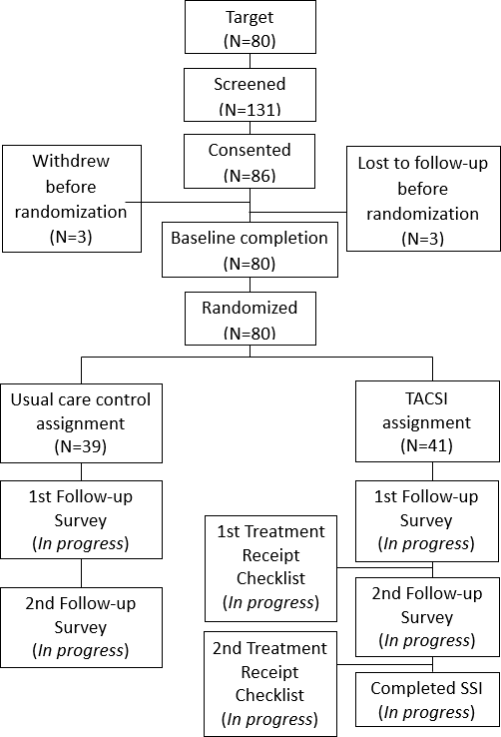
TACSI study flowchart. SSI: semistructured interviews; TACSI: TBI (traumatic brain injury)-AD/ADRD (Alzheimer disease and related dementias) caregiver support intervention.

Study enrollment began in June 2023. Target enrollment (n=80) was reached in November 2024. Final data collection was completed in June 2025 (Figure 3). Participants were administered consent procedures via their preferred modality: (1) a coordinator read a consent script and obtained caregiver consent verbally via telephone; (2) the caregiver reviewed a web-based consent form and consented via an electronic signature; or (3) the caregiver reviewed a mailed hard copy of the consent form prior to providing telephone consent. Interested caregivers were encouraged to ask questions prior to providing consent for study participation; participants were sent a copy of the completed consent form for their records.

Following consenting procedures, participants were administered a baseline survey; baseline surveys included demographic items for the participating caregiver and the person they care for, as well as a battery of quantitative survey measures. Table 1 displays the SPIRIT timeline [30].

**Table 1.** SPIRIT (Standard Protocol Items: Recommendations for Interventional Trials) timeline of enrollment, intervention, and measures.

Activity or assessment	Prestudy	Baseline survey (t_0_)	Randomization	Follow-up survey (t_1_) 3-months post- t_0_ completion	Follow-up survey (t_2_) 3-months post-t_1_ completion	End of study
Eligibility screen	✓					
Informed Consent	✓					
Outcome measures^a^						
Modified Caregiver Appraisal Scale [36] measuring Perceived burden, Caregiver relationship satisfaction, Caregiving ideology, and Caregiving mastery		✓		✓^g^	✓^g^	
Psychological Distress: The Center for Epidemiological Studies-Depression Scale [37]		✓		✓	✓	
TBI-CareQOL^c^ Feelings of Loss – Self – Short Form 6a [38]		✓		✓^g^	✓^g^	
TBI-CareQOL Feelings of Loss – Person with TBI – Short Form 6a [38]		✓		✓^g^	✓^g^	
TBI-CareQOL Feeling Trapped – Short Form 6a [38]		✓		✓^g^	✓^g^	
TBI-CareQOL Caregiver-Specific Anxiety – Short Form 6a [38]		✓		✓^g^	✓^g^	
TBI-CareQOL Caregiver Strain – Short Form 6a [38]		✓		✓^g^	✓^g^	
TBI-CareQOL Caregiver Vigilance – Short Form 6a [38]		✓		✓^g^	✓^g^	
TBI-CareQOL Emotional Suppression – Short Form 6a [38]		✓		✓^g^	✓^g^	
TBI-CareQOL Family Disruption – Short Form 3a [38]		✓	✓	✓^g^ Omit if bereaved	✓^g^ Omit if bereaved	
Health Services Service Use		✓		✓	✓^d^ Omit if previously completed post-bereavement	
Community-Based Services Use [39]		✓		✓	✓^d^ Omit if previously completed post-bereavement	
COPE [40]		✓		✓	✓	
Interpersonal Support Evaluation List [41]		✓		✓	✓	
Caregiver Self-Efficacy Scale [42]		✓		✓^g^	✓^g^	
Activities of Daily Living/Instrumental Activities of Daily Living (ADL/IADL) [43, 44]		✓		✓	✓^d^	
Revised-Memory and Behavior Checklist [45]		✓		✓^g^	✓^g^	
Intervention						
Caregiver support intervention			✓			
Usual care control assignment			✓			
Treatment Receipt Checklist				Treatment group only	Treatment group only	
Semistructured Interview^e^						Select treatment group participants

a More information about the outcome measures can be found in the Multimedia Appendix 1 [34,35,46-53].

b Omit if bereaved.

c TBI-CareQOL: Traumatic Brain Injury Caregiver Quality of Life.

d Omit if previously completed post-bereavement.

e Interview questions can be found in the Multimedia Appendix 2.

#### Randomization Procedures

After baseline survey completion, participating caregivers were randomly assigned on a 1:1 basis to participate in either (1) the TACSI program treatment group or (2) the usual care control group (the latter continued routine care through their HCS). The random assignment schedule was created digitally by a staff member involved in the study solely for this task, via Research Randomizer. Participants had equal chances of being assigned to either the treatment or control group. The assignment schedule was input into a series of sequenced envelopes to be opened in order at the time of randomization (following a participant’s baseline survey completion).

The study coordinator informed participants about the results of their randomization to either the TACSI treatment group or the usual care control group via phone, email, or both. Coordinators also alerted the TACSI study coaches, who divided the participant caseload based on workload and availability. Those in the control group were given the opportunity to request resources or referrals during their randomization notification and as a part of the survey completion thank you procedures in order to provide support and bolster retention.

#### Intervention

##### Coaching Sessions

###### Overview

After randomization, the assigned TACSI coach contacted the caregiver assigned to the treatment group to offer introductory information, including a description of the semistructured program (eg, design, length, and frequency of sessions) and to determine caregiver preferences for participation (eg, modality of delivery).

The TACSI program is a 6-session semistructured psychosocial and psychoeducational program, developed to increase caregiving skills and strategies and provide support to improve mood, reduce stress, and increase caregiver self-efficacy. TACSI provides individual coaching and ad hoc support to caregivers. Intervention sessions are conducted on a weekly basis, as schedules permit. Sessions typically last from 45 minutes to an hour and a half, depending on discussion needs and participant preferences. Sessions are held remotely via telephone or secure video conferencing (ie, Zoom) based on participant requests. Table 2 includes descriptions of TACSI’s mode of delivery, frequency, and other delivery characteristics adapted from the intervention taxonomy of Schulz et al [46].

The initial TACSI session includes an informal interview to build rapport and allow the coach to learn about the caregiver and care recipient, identifying critical stressors and priorities for the intervention. All subsequent sessions include a brief check-in for caregivers to share updates of their previous week and ascertain the most relevant focus of the session, followed by a discussion of pertinent module content. The conclusion of each session may include discussion of self-care plans for the upcoming week, determining goals or homework, and scheduling the next session, if applicable. The order of delivery of TACSI modules 2 through 5 is intentionally flexible to respond to the priorities and needs of the participant. The sixth session serves to discuss any unexplored intervention content, review previously presented material, and address any remaining caregiver questions.

A TACSI intervention manual provided an overarching structure and guide to facilitate the coach’s intervention delivery. The semistructured nature of the program allows coaches the flexibility to tailor the intervention based on the caregiver’s needs and priorities. All topics in the manual are offered during the intervention, but the depth of exploration, if any, is determined by the caregiver. Further, tailoring and personalization of the session content and focus are emphasized by the caregiver guiding the selection of topics to discuss and deciding when to discuss them during the intervention.

**Table 2.** TBI-AD/ADRD^a^ caregiver support intervention (TACSI) delivery characteristics.

Dimension	Options
Method of caregiver/coach contact	Telephone Secure video conferencing (ie, Zoom) Email Mail
Intervention materials	Coaches: Treatment manual Computer with word processing for session notes Internet access (for secure video conferencing, ie, Zoom; resource identification/use) Printer to share resources if needed Caregivers: Computer, phone, or both to access remote intervention
Delivery location	Remote delivery via: Telephone Secure video conference calls
Schedule: duration and intensity	Session types: Six weekly TACSI sessions (approximately 60 minutes each) Ad hoc consultation as needed (approximately 45 minutes) Participants could voluntarily withdraw from intervention at any time. Time needed for documentation (approximately 30 minutes post-session): Session dates Session length Number of ad hoc sessions Length of ad hoc sessions Session notes
Scripting/tailoring of intervention	Semistructured intervention based on treatment manual General guidelines and some language provided; elaboration and discussion encouraged Topics specified; coach tailoring of which topics to discuss and when, based on caregiver priorities and needs Personalization emphasized based on caregiver and care recipient background, experiences, and abilities
Sensitivity to participant characteristics	Personalization of session content/topics (see Scripting/Tailoring of Intervention) Language preferences, literacy, visual supplements/augmented communication have not been incorporated or requested by participants
Interventionist qualifications, training, and experience	Bachelor’s degree or higher Experience working with individuals with dementia and their caregivers Training relevant to understanding the treatment manual and ongoing support for accurately implementing the intervention: Thorough step-by-step review of the treatment manual Regular coaching meetings (problem-solve questions and concerns, share available resources, etc) Role play intervention material (Consider having trainees shadow/listen to sessions or mentors observe and provide feedback on trainee’s sessions) Competence in remote intervention delivery Ability to build and maintain rapport
Adaptability: extent to which intervention can be modified	What: Ad-hoc sessions Session length Caregiver location Mode of delivery (telephone vs secure video conference) Session Content When: Any time while enrolled in study Why: Participant request Participant availability Participant preference Coach judgment
Treatment implementation: Delivery: documentation of interventionist compliance to intended treatment and modifications Receipt: extent to which processes are implemented by participant and goals are met Enactment: extent to which knowledge and skills acquired during treatment are applied in real-world settings outside of treatment	Documentation: Number and duration of sessions Content delivered Participant Data Collection: Treatment receipt checklist survey responses Participant self-report during sessions Coach appraisal of participant understanding, acquired skills, caregiving self-efficacy, engagement, and use of supports (recorded in coaching notes) Semistructured interviews conducted with purposively sampled primary caregivers at conclusion of participation
Content and goals	
Treatment content strategies: specific strategies aimed at improving outcomes	Psychoeducation/didactic instruction Personalized resource identification and provision Facilitation of family and social support and involvement Effective communication and conflict resolution skills, including advocacy Skill-building techniques and problem-solving techniques Stress management techniques and relaxation exercises Processing and emotional support, including for guilt and grief Active coping strategies
Mechanisms of action: key processes, goals, or mediators of desired treatment outcomes	Improved knowledge of dementia Improved engagement with care recipient, family, and health care team Increased awareness of resources and supports Improved social and family support Improved communication and conflict resolution skills Increased problem-solving skills Increased prioritization and engagement in self-care and stress management Increased caregiving self-efficacy Increased caregiving competence

a TBI: traumatic brain injury; AD/ADRD: Alzheimer disease and related dementias.

In each session, coaches aim to establish and maintain a therapeutic rapport with the caregiver while providing a safe, neutral third-party environment to identify, explore, and discuss their stressors. Coaches engage in thoughtful conversation, providing psychoeducation and resources to support each caregiver. They provide individualized constructive guidance and feedback to help caregivers address their unique needs, respond to challenging situations, and use newly learned skills. Coaches also have the flexibility to tailor the length of sessions and to include topics outside of the manual to meet the participant’s needs. After each meeting, coaches record session notes, detailing key caregiving issues, relevant family dynamics, the caregiver’s reaction to the session, and anticipated content for the subsequent session.

TACSI is a multicomponent program combining psychosocial support with psychoeducational and skills training. Multicomponent interventions have been associated with improving caregiving outcomes [47,48]. TACSI was developed by one of the study coaches (RB) by modifying a well-received and tested intervention, the residential care transition module, created by the UMN team for a previous dementia caregiver support intervention [49-52]. Moreover, the residential care transition module was modeled after the New York University Caregiver Intervention, which has been found to improve dementia caregivers’ well-being, depressive symptoms, and their appraisal of behavioral issues in their care recipients [53-57]. These interventions were grounded in the Stress Process Model [58].

Modifications for TACSI included adding TBI-specific content, such as education on how a TBI may affect the brain and behavior, managing TBI-related concerns, and resources for caring for individuals with TBI. The TACSI program also featured an increased number of approaches for managing negative or anxious thoughts and additional new topics such as how to work effectively with the care recipient’s health care team, how to ask for and accept help, and how to navigate changes in partner intimacy. Additionally, coinvestigator JF, an expert on TBI caregiving, reviewed and edited the intervention manual, providing feedback and recommended resources. Table 3 provides an overview of the TACSI program content.

**Table 3.** TBI-AD/ADRD^a^ caregiver support intervention program content.

Session	Content
Session 1:	Module 1 (caregiver interview): to explore the caregiver’s journey (eg, individuals involved, roles, stressors, strengths/weaknesses of care, etc) to guide the intervention.
Modules 2-5:	Module 2 (increasing understanding of dementia and traumatic brain injury and building awareness of supports and services): (1) to provide psychoeducation TBO and dementia, as well as impact on daily life (brain, cognition, personality, and behavior), (2) to discuss local/national supports tailored to individual participants’ needs, and (3) to introduce/set SMART^b^ goals. Module 3 (supporting emotional health and well-being): (1) to explore experiences of guilt and grief and (2) to increase the understanding of stress and coping strategies. Module 4 (encouraging realistic expectations and adjusting to lifestyle changes): (1) to explore roles and expectations and (2) to share activity ideas. Module 5 (effectively working with a health care team/enhancing communication): (1) to review tips for navigating and partnering with the health care team and (2) to discuss conflict resolution.
Session 6:	Module 6 (review of intervention topics and discussion of wrap-up questions): (1) to wrap up/highlight any intervention content and (2) to review wrap-up questions assessing caregiver adjustment, strengths, and challenges.

a TBI: traumatic brain injury; AD/ADRD: Alzheimer disease and related dementias.

b SMART: Specific, Measurable, Achievable, Relevant, Time-Bound.

**Figure 4.**
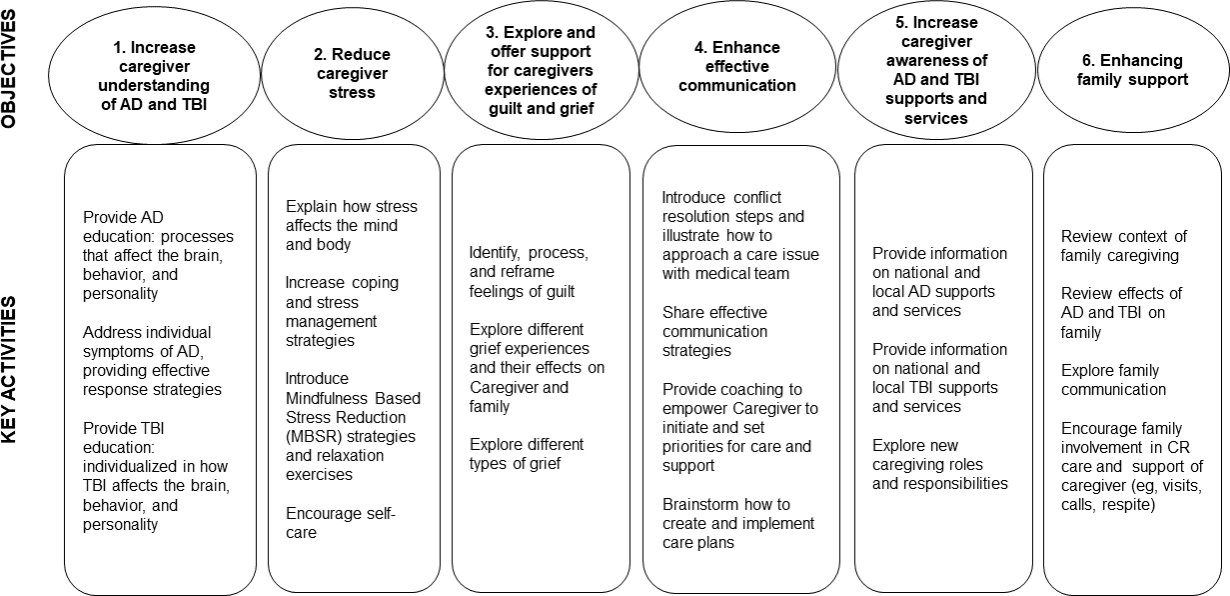
TBI (traumatic brain injury)-AD/ADRD (Alzheimer disease and related dementias) caregiver support intervention (TACSI) map. AD: Alzheimer disease.

Intervention sessions focus on exploring the participant’s caregiving experience, including the supports and strategies they currently use, while determining and subsequently sharing skills, approaches, and new information they likely will benefit from acquiring. A key objective for each session is to build the caregiver’s resources in order to alleviate primary subjective stressors, increase caregiver confidence, and enhance awareness and use of informal and formal supports and services. Intervention content and coaching strategies were purposefully selected to positively impact primary (caregiver perceived burden, relationship satisfaction, ideology, and mastery) and secondary outcomes (table of measures is provided in Multimedia Appendix 1 [34,35,46-53]). The following 4 domains serve as the foundation for achieving the 6 objectives and affiliated key activities as detailed in the intervention map (Figure 4).

###### Dementia and TBI Education (Addressing Objective 1)

To increase understanding of AD/ADRD and TBI, coaches share how TBI and dementia affect the brain and how that may impact behavior, personality, and cognition. This education provides an explanation and foundation for the caregiver to understand why the relative may be functioning as they are and how they may be impacted in the future. Learning that their care recipient’s behavior is not an intentional choice, but a result of the injury or disease process, can help reduce the annoyance some caregivers may experience. It also helps them to have appropriate expectations and to make plans for the future. In addition, the intervention shares approaches that help to prevent and better manage frustrating behavior (eg, aggression, wandering, hygiene avoidance). Education also focuses on processing and addressing uncomfortable role changes that the caregiver may be experiencing, including adjusting to new responsibilities and changes in spousal intimacy.

###### Promoting Stress Management and Well-Being (Addressing Objectives 2 and 3)

The intervention aimed to help improve mood and manage stressors. Exploration of the caregiver’s experiences with guilt and grief sought to help identify sources of negativity and subsequently, process, validate, normalize, and reframe those negative emotions. Stressors were identified, including those related to caregiving, as well as outside sources of stress. Stress management techniques, including relaxation exercises and problem-solving strategies, such as breaking a larger problem into smaller tasks and setting specific, measurable, achievable, relevant, and time-bound goals, were explored. The caregiver’s experience with loneliness and social isolation was explored, along with the importance of self-care and social engagement.

###### Enhancing Communication (Addressing Objective 4)

Coaches focused on providing a range of communication skills and strategies for caregivers to use with their care recipients, families, health care teams, and others. Through psychoeducation on the tenets of effective communication and the sharing of conflict resolution strategies, the intervention aims to help caregivers convey their perspective, gain understanding and support from others, and advocate for their care recipient. Coaches also offer approaches on how to best engage with a health care team, adapted from the Health Resources and Services Administration’s “Working with a Healthcare Team” module [59]. Additionally, coaches share strategies to improve communication and engagement with the care recipient.

###### Building Awareness of Supports (Addressing Objectives 5 and 6)

Caregivers learn about the local and national supports and services that can meet their unique needs. Provision of these personalized local and national formal supports is complemented by the identification of sources of informal support, such as family, friends, and neighbors. Once these informal sources of support are identified, coaches process caregiver reservations about asking for assistance, provide guidance, and encourage caregivers to ask for and accept offers of help and respite.

### Ad Hoc Sessions

Ad hoc sessions, delivered via telephone, secure web conferencing, or email, were available at any time between sessions and following intervention completion. Caregivers could schedule a standing ad hoc session or request impromptu ad hoc coaching. Ad hoc sessions and email consultations were unstructured and designed to address any caregiver concerns in a timely manner.

### Interventionist Characteristics

The program is designed to be delivered by a qualified coach based on education or training. Recommended qualifications include: (1) a Bachelor’s degree or higher in a health or wellness-related field; (2) relevant experience working with people with memory loss or their caregivers; (3) experience or proficiency in remote intervention delivery, particularly having the ability to establish rapport without being in person; and (4) completion of TACSI training based on the intervention manual. See Table 2 for additional interventionist characteristics [46].

TACSI coaches were 2 doctorally educated staff (DNP and PhD in clinical psychology). Both have personal and professional experience with individuals with dementia and their caregivers, as well as experience with remote intervention delivery. The coaches worked together previously on ADRD caregiving studies. Similar procedures were used in this study as in the previous intervention studies.

The coach-developed (RB) intervention includes a manual that specifies the procedures for delivering the intervention and details module content. The manual serves as a guide on intervention structure, delivery processes, and session content. Coach training consisted of a thorough step-by-step review of the treatment manual, including discussion of appropriate question prompts, how to start topic discussions, and how to tailor the content to the individual participant. Coaches meet regularly to discuss the intervention, problem-solve any concerns, and share information and newly discovered resources. Coinvestigator JF, a MVAHCS staff psychologist, was present at weekly meetings throughout study recruitment and was available to confer with coaches on any issues as needed.

### Data Collection and Measures

#### Overview

Data collection for both treatment and control groups was conducted via surveys. Additional data were collected from treatment group caregivers via TRCs and semistructured interviews.

#### Surveys

Caregivers received a baseline survey collecting information on sociodemographics, context of care, and outcome measures. The baseline survey contained sociodemographic measures for both the caregiver and care recipient (date of birth, gender, racial background, marital status, education level, and household income). Context of care variables, such as geographic location, caregiver work status, health conditions, and relationship to care recipient were assessed. Additional variables included year and severity/type of TBI and AD/ADRD diagnoses with concurrent symptoms, care recipient Veteran and Medicaid status, and living arrangement.

Two follow-up surveys were administered by a research assistant blinded to participant group assignment from randomization through the end of analysis. The first follow-up (T1) was administered at 3 months post completion of the baseline survey; the second follow-up was administered 3 months after completion of the 3-month survey (T2). If a care recipient passed away during the study, modified bereavement surveys were administered at follow-up.

Three options for completing surveys were offered: online via Qualtrics, a mailed survey, or over the phone with study staff. The specific measures selected for assessment have strong psychometric properties; Table 1 displays outcome measures administered by time point and survey type. Primary outcome measures assessed perceived burden, caregiver relationship satisfaction, caregiving ideology, and caregiving mastery using the Modified Caregiver Appraisal Scale [36]. Secondary outcome measures evaluated caregiver well-being via the Center for Epidemiological Studies-Depression Scale [37] and multiple TBI caregiver quality of life [38] subscales assessing feelings of loss, feeling trapped, caregiver anxiety, strain, vigilance, emotional suppression, and family disruption. Secondary outcome measures also evaluated caregiver resource usage, including hospital and residential care service use and community-based services such as housekeeping [39], caregiver personal resources (coping style [40], interpersonal supports [41], and self-efficacy [42]), ADLs/instrumental activities of daily living [43,44], and care recipient behaviors [45].

Adverse events were defined prior to the onset of the study, and standardized procedures were determined to address these events, if encountered. At the 3 and 6 month follow-up timepoints, participants were asked: “In the last three months, since your last survey, was there any new or worsening health problem that caused you to be unable to perform your daily routine (ie, not go to work or volunteer), seek medical care (ie, go see your doctor, go to the ER or hospital), or take a new medication?” Those medical events were documented as adverse events. In addition, during each intervention session, coaches inquired into and addressed participant health and other concerns, as applicable.

Staff provided up to 4 reminders to complete each survey via phone or email. If a follow-up survey was not completed, subsequent surveys were still sent unless the participant withdrew from the study. Staff provided a thank-you phone call or email after completion of each survey.

#### Treatment Receipt Checklist

Within 2 weeks of the completion of their follow-up surveys (T1, T2), treatment group caregivers were sent an additional survey by an unblinded staff member to evaluate treatment receipt via a brief TRC, as well as assess the acceptability, appropriateness, and feasibility of the intervention. Unblinded staff provided reminders to complete the TRCs; thank you emails or calls were completed following survey completion.

The TRC is a 13-item survey evaluating participant receipt of various components of the intervention using 12 Likert scale items (1=strongly disagree to 5=strongly agree, with an option for not applicable/does not apply to me). An open-ended question inquiries about how TACSI was or was not helpful (Wilson et al [29] provides TRC items).

Acceptability, appropriateness, and feasibility of the intervention were evaluated using the acceptability of intervention measure (AIM), the intervention appropriateness measure (IAM), and the feasibility of intervention measure (FIM) [60]. Each measure consists of 4 items using a 5-point Likert scale (1=completely disagree; 5=completely agree). These measures demonstrate strong validity, high internal consistency (AIM, α=.85; IAM, α=.91; and FIM, α=.89), and high test-retest reliability (AIM=0.80, IAM=0.73, FIM=0.88).

#### Bereavement or Residential Placement

If the care recipient passed away during the study, the participating caregiver was offered condolences by the staff. Pending their willingness to continue the study, these caregivers were administered modified bereavement surveys at follow-up. If the caregiver was in the treatment group, they no longer participated in standard TACSI sessions. However, the interventionist offered optional ad hoc sessions focused on the participant’s needs, integrating TACSI content only if relevant. Depending on the number of study time points remaining, either one or two bereavement surveys would be administered, both omitting measures no longer applicable, as shown in Table 1.

In the case of a care recipient’s nursing home admission, the caregiver could continue study involvement (TACSI intervention and routine follow-up surveys) as usual.

#### Semistructured Interviews

Twenty participants in the treatment group were purposely selected to participate in an audio-recorded semistructured interview to provide qualitative data regarding the TACSI program. Staff selected participants who rated TACSI with higher and lower average scores on the TRC and measures assessing the intervention’s appropriateness, acceptability, and feasibility (see above). Additionally, as the sample allowed, the study team made an effort to select participants with varied characteristics: relationship to care recipient (partner, adult child), severity of condition (TBI-AD/ADRD), and racial/ethnic identity. Participants could complete the semistructured interview via phone or secure video conferencing (Zoom). Multimedia Appendix 2 contains the interview guide.

#### Coach Documentation

Throughout each caregiver’s time in the study, coaches documented any participant contact for treatment and control participants: dates, method of contact, length of contact, and general topics of discussion. Session notes were also completed by coaches, documenting key issues, family dynamics, participant response or engagement with session content, and plans for the next session’s content.

### Analysis

Quantitative analysis will include descriptive statistics, such as frequency tables and means, to describe sample characteristics and TBI-AD/ADRD caregiver outcomes. Enrollment and recruitment data, such as the number of contacts or referrals by site, reasons for exclusion, and retention, will be summarized. Distribution/normality and missing data will also be examined.

The number of TBI-AD/ADRD caregivers enrolled to address the study hypotheses (n=80) was determined using power analysis procedures that relied on Wänström’s Monte Carlo simulations [61]. A final sample of 72 TBI-AD/ADRD caregivers was found sufficient for detecting a medium effect size of 0.5 at a statistical power level of 0.8, while adjusting for 10% loss to follow-up and for multiple potential statistical differences between treatment and control groups at baseline. In order to evaluate whether a full-scale randomized controlled design is feasible and appropriate, we will conduct paired *t* tests to determine whether the TACSI treatment and control groups are statistically (*P*>.05) equivalent across all baseline outcomes and TBI-AD/ADRD caregiver covariates. Multilevel regression analyses of outcome and growth curve modeling will be used to compare rates of change between the treatment and control groups in primary subjective stressors and other caregiver outcomes. The primary independent variable will indicate random assignment into the TACSI treatment or control group. If any sample characteristics or outcomes vary significantly at baseline between the treatment and control groups, the baseline values will be included as covariates. In order for the intercept effect to be a main effect and to have a treatment*time interaction effect, the baseline value of each outcome will be a covariate, and time will be centered at 3 months after the baseline survey. We will explore whether treatment participants experienced statistically significant (*P*<.05) declines in primary subjective stress, increased well-being, and other changes in TBI-AD/ADRD caregiver outcomes when compared with controls over the study period. Further, Cox proportional hazard models will be run with the events including admission to a nursing home, assisted living, similar residential living situation, and emergency department and overnight hospital stays.

We will calculate means and create frequency tables to evaluate AIM, IAM, and FIM measures and TRC at T1 and T2. We will determine if 80% of treatment caregivers found the intervention appropriate, feasible, and acceptable at the 2 follow-up timepoints (T1 and T2).

Post hoc analyses will also be conducted. Specifically, to examine the effects of TACSI use on changes in primary subjective stress and caregiver well-being, intervention session data regarding variations in use of TACSI, such as frequency and duration of counseling sessions, will also be included in the growth curve models.

The qualitative analysis will use reflexive thematic content analytic techniques as described by Morse, Moser, and others [62-66]. The semistructured interviews and open-ended comments from the TRCs will comprise the qualitative data. The research staff will read the qualitative data to familiarize themselves with the data and make changes to the Phase I codebook accordingly. Staff will review and discuss coding assumptions before making any revisions to the codebook. This qualitative analysis will help determine why TACSI did or did not meet the needs of TBI-AD/ADRD caregivers.

The mixed methods analysis aims to provide empirical context for the participants’ perceptions of TACSI. A sequential mixed methods sampling approach was used in selecting treatment participants for the semistructured interviews. This will enable research staff to better understand participants’ perceptions of TACSI’s usefulness while also linking qualitatively derived themes with the outcome data. Specifically, the empirical outcome data will be sorted by qualitative themes that appeared across the 2 subgroups of the semistructured interview sample (those who rated TACSI as highly acceptable, feasible, and useful in the TRCs vs those who rated TACSI poorly on the same measures). This mixed methods approach will allow us to determine if certain themes appear more often for one subgroup than the other subgroup, or if the subgroups use TACSI recommendations differently.

### Data Sharing

To facilitate data preservation and sharing following project completion, the study protocol includes a plan to deposit quantitative survey study data into a centralized repository. Pending regulatory recommendations and availability at the time of post-study submission, the team has focused data preparation efforts on procedures developed by the Federal Interagency Traumatic Brain Injury Research Informatics System, developed by the National Institute of Health in collaboration with the Department of Defense. Alternatively, the Data Repository for University of Minnesota (a data repository located at UMN) or a similar resource will be used.

### Dissemination Plan

Results of this study will be shared with participants via a one-page summary and posted to the team’s website. The team will publish the results in academic journals and potentially present at gerontological conferences and community sites, including at TBI and dementia-focused organizations.

## Results

Figure 3 depicts participants’ flow through the study. All participants have been recruited. Enrollment of 80 participants began in June 2023. Survey data collection completed in late June 2025. Figure 3 shows the general flow of participants throughout the study.

## Discussion

Within the dementia care intervention research literature, care recipient condition (eg, “Alzheimer’s disease or a related dementia”) is often conceptualized as a solitary challenge influencing care recipient symptoms, care demands, and ultimately, caregiver distress and well-being. However, persons living with ADRD often experience other serious conditions either concurrently (eg, “mixed” dementia) or in instances where another condition’s onset may have predated the onset of ADRD, such as TBI [1,67,68]. With the potential complexities for caregivers in managing symptoms and care demands in these situations, we developed TACSI as a potential support resource for family members and other unpaid individuals to assist in navigating the TBI/ADRD care context.

With the exception of concurrent efforts to adapt the evidence-based REACH intervention in the Veterans Administration HCS, we know of no other caregiver intervention model that is tailored to the needs of caregivers of individuals with TBI/ADRD. Given how TBI is a consistent risk factor for ADRD (see above), creating a psychosocial and psychoeducational program that targets increased understanding, stress reduction, guilt and grief management, effective communication, and awareness of support locally and within the family may help to address an important gap in care for people living with TBI/ADRD and those who support them.

This pilot randomized controlled evaluation, along with the mixed methods experimental design, will yield important insights as to the readiness of TACSI to proceed to a larger-scale randomized controlled evaluation to demonstrate efficacy, as well as how and why the intervention is beneficial to TBI/ADRD caregivers [30,34]. The latter is particularly crucial in the developmental life cycle of a behavioral intervention, as the use of qualitative data to identify mechanisms of action/benefit may help to guide their measurement and testing in subsequent efficacy trials and help to establish the essential aspects of an intervention that must be maintained as it progresses from efficacy to later effectiveness testing and, ultimately, successful dissemination/implementation.

Across chronic disease contexts, and particularly in TBI and ADRD, the ramifications of the disease trajectory extend far beyond the person living with one or both of these conditions. To achieve optimal care for persons with TBI/ADRD, a socioecological perspective is warranted, where medical and psychosocial support target both the person living with these conditions and those involved in their care, most often family members. The TACSI program adopts such a perspective in its focus, intervention targets, and outcomes. The use of a mixed methods experimental intervention design will further facilitate the: 1) piloting of the RCT design to establish its feasibility; and 2) identification of how and why the intervention yields benefits for caregivers of individuals with TBI/ADRD, establishing a strong scientific foundation from which to proceed to a larger scale RCT design to demonstrate efficacy.

When considering the design, testing, and (it is hoped) successful dissemination and implementation of TACSI, several key issues are essential to note. One aspect of TACSI that helps to facilitate its translation and dissemination is its remote/telehealth delivery. A potential implementation barrier will be to scale the program so that interventionists can deliver the protocol and not require advanced professional training and credentials (as the current TACSI interventionists hold). Moreover, if the latter translational objective is achieved, the development of an asynchronous training protocol so that interventionists can easily obtain the skills and certification needed to deliver TACSI will require development and refinement in order for the intervention to achieve its full implementation/dissemination potential. Another key aspect of our design that will facilitate subsequent efficacy and effectiveness testing is the use of mixed methods to identify mechanisms of action (in addition to the ones already hypothesized as part of the TACSI via the Stress Process Model; please see above) that will underscore the essential elements of the intervention that should be maintained if/when the intervention is adapted for effectiveness testing and dissemination/implementation in heterogeneous clinical and community settings.

The prevalence of mixed dementia, where persons diagnosed with Alzheimer disease are found to have multiple causes of dementia (eg, Alzheimer disease and TBI), is present in half or more of individuals living with ADRD [1,67,68]. Given the long-established challenges of dementia care for unpaid family members, friends, and others, there is a pressing need to develop support programs and resources for caregivers of persons with potentially concurrent causes of dementia. It is hoped that TACSI can fill this gap for caregivers of persons with TBI/ADRD.

## Supplementary material

10.2196/81125 Multimedia Appendix 1 Survey measures.

10.2196/81125 Multimedia Appendix 2 Semistructured interview guide.

10.2196/81125 Checklist 1 SPIRIT 2025 checklist.

## Abbreviations

AD/ADRD: Alzheimer Disease/Alzheimer Disease and Related Dementias
ADL: activities of daily living
AIM: acceptability of intervention measure
FIM: feasibility of intervention measure
HCS: health care system
HIPAA: Health Insurance Portability and Accountability Act
IAM: intervention appropriateness measure
*ICD*: *International Classification of Diseases*
*ICD-10*: *International Statistical Classification of Diseases, Tenth Revision*
MVAHCS: Minneapolis Veterans Affairs Health Care System
RCT: randomized controlled trial
REACH: Resources for Enhancing Alzheimer’s Caregiver Health
SPIRIT: Standard Protocol Items: Recommendations for Interventional Trials
TACSI: TBI-AD/ADRD Caregiver Support Intervention
TBI: traumatic brain injury
TRC: treatment receipt checklist
UMN: University of Minnesota
