# Supplementary material for: The TBI-AD/ADRD Caregiver Support Intervention (TACSI): Protocol of a Pilot Randomized Controlled Trial Evaluation of a Remote Intervention for Family Caregivers
Source: JMIR Res Protoc. 2026 Mar 17;15:e81125. doi: 10.2196/81125 (PMC12994761; doi:10.2196/81125)
Supplement: Multimedia Appendix 1 [file resprot-v15-e81125-s001.docx]

| **Type of Measure/Outcome** | **Description** |
| --- | --- |
| **Primary Outcomes** | |
| Primary subjective stressors | Measuring Perceived burden, Caregiver relationship satisfaction, Caregiving ideology, Caregiving mastery via the Modified Caregiver Appraisal Scale assesses agreement with 35 statements on a 5-point Likert scale ranging from strongly disagree (1) to strongly agree (5).^1^ |
| **Secondary Outcomes: Caregiver Well-Being** | |
| Psychological Distress: The Center for Epidemiological Studies-Depression Scale | 20-item measure assessing the number of days participants had behaved or may have felt in the past week on a 4-point scale (5-7 days, 3-4 days, 1-2 days, or less than 1 day).^2^ |
| TBI-CareQOL Feelings of Loss – Self – Short Form 6a | 6-item scale assessing feelings of sorrow, mental suffering or distress over changes that the caregiver has personally experienced related to the TBI on a 5-point scale ranging from never (1) to always (5).^3^ |
| TBI-CareQOL Feelings of Loss – Person with TBI – Short Form 6a | 6-item scale assessing feelings of sorrow, mental suffering, or distress related to changes in the person with TBI (including loss of abilities, loss of potential/future, and changes in behaviors/personality) on a  5-point scale ranging from never (1) to always (5).^3^ |
| TBI-CareQOL Feeling Trapped – Short Form 6a | 6-item scale assessing the feelings that one is unable to go places or do things due to caregiving responsibilities on a 5-point scale ranging from never (1) to always (5).^3^ |
| TBI-CareQOL Caregiver-Specific Anxiety – Short Form 6a | 6-item scale examining feelings of anxiety/worry related to the caregiver role and concern/worry about the behavior and well-being of the person with TBI on a 5-point scale ranging from never (1) to always (5).^3^ |

| TBI-CareQOL Caregiver Strain – Short Form 6a | 6-item scale examining feelings of being overwhelmed, stressed, self-defeated, downtrodden, and beat-down related to the caregiver role on a 5-point scale ranging from never (1) to always (5).^3^ |
| --- | --- |
| TBI-CareQOL Caregiver Vigilance – Short Form 6a | 6-item scale assessing caregiver-specific feelings of hyperarousal or vigilance related to concerns about the emotional and behavioral status of the person with the TBI on a 5-point scale ranging from never (1) to always (5).^3^ |
| TBI-CareQOL Emotional Suppression – Short Form 6a | 6-item scale assessing caregivers' hiding or suppressing of negative feelings while acting in the caregiver role on a 5-point scale ranging from never (1) to always (5).^3^ |
| TBI-CareQOL Family Disruption – Short Form 3a | 3-item scale assessing how caring for an individual with a TBI interferes with family life on a 5-point scale ranging from never (1) to always (5).^3^ |
| **Secondary Outcomes: Caregiver Resource Utilization** | |
| Service Use | Five Yes/No questions about admission to nursing homes, assisted living, other residential care settings, and overnight hospital stays and emergency room visits. Also asks about the number of nights or visits in the past 3 months. |
| Community-Based Service Use | Respondent indicates their or CR use of 14 formal/paid services over the past 3 months, with the 15th option as a write-in.^4^ |
| **Secondary Outcomes: Caregiver Personal Resources** | |
| COPE | A selection of 24 items from three domains of COPE (problem-focused coping, emotion-focused coping, and avoidant coping) assessing the coping style of caregivers to stressors related to care provision. Response options range 1 (‘I usually don’t do this at all’) to 4 (‘I usually do this a lot’).^5^ |
| Interpersonal Support Evaluation List | 16-statements where respondent indicates if statement was either definitely true, probably true, probably false, definitely false for them.^6^ |
| Caregiver Self-Efficacy Scale | 8-item measure assessing caregiver self-efficacy. Responses ranged from 1 (not certain at all) to 10 (very certain).^7^ |
| **Secondary Outcomes: Primary Objective Stressors** | |
| Activities of Daily Living/Instrumental Activities of Daily Living (ADL/IADL) | 12 items on ADLs/IADLs - asking if CR needs “no help, some help, a lot of help, or unsure” if the CR wanted to do the activities.^8,9^ |
| Revised-memory and behavior checklist | 24 items assessing presence of behaviors (yes/no) and how much it has bothered the caregiver on a 4-point scale, ranging from not at all (0) to extremely(4).^10^ |

**References**

1. Gallo JJ, Lee SY. Mixed methods in behavioral intervention research. In: Gitlin LN, Czaja SJ, eds. Behavioral Intervention Research: Designing, Evaluating, and Implementing. Springer Publishing; 2016:195-212.
2. Abbott JH. The distinction between randomized clinical trials (RCTs) and preliminary feasibility and pilot studies: what they are and are not. J Orthop Sports Phys Ther. 2014;44(8):555-558. doi:10.2519/jospt.2014.0110
3. Schulz R, Czaja SJ, McKay JR, Ory MG, Belle SH. Intervention Taxonomy (ITAX): Describing Essential Features of Interventions (HMC). Published online 2010.
4. Schulz R, O’Brien A, Czaja S, et al. Dementia Caregiver Intervention Research: In Search of Clinical Significance. Gerontologist. 2002;42(5):589-602.
5. Sörensen S, Pinquart M, Duberstein P. How effective are interventions with caregivers? An updated meta-analysis. Gerontologist. 2002;42(3):356-372. doi:10.1093/geront/42.3.356
6. Gaugler JE, Reese M, Sauld J. A Pilot Evaluation of Psychosocial Support for Family Caregivers of Relatives with Dementia in Long-Term Care. Res Gerontol Nurs. 2015;8(4):161-172. doi:10.3928/19404921-20150304-01
7. Albers EA, Birkeland RW, Louwagie KW, et al. A Qualitative Analysis of Mechanisms of Benefit in the Residential Care Transition Module: A Telehealth Intervention for Caregivers of Relatives With Dementia Living in Residential Long-Term Care. Inquiry. 2023;60:469580231217981. doi:10.1177/00469580231217981
8. Gaugler JE, Statz TL, Birkeland RW, et al. The Residential Care Transition Module: a single-blinded randomized controlled evaluation of a telehealth support intervention for family caregivers of persons with dementia living in residential long-term care. BMC Geriatrics. 2020;20(1):133. doi:10.1186/s12877-020-01542-7
9. Gaugler JE, Birkeland RW, Albers EA, et al. Efficacy of the residential care transition module: A telehealth intervention for dementia family caregivers of relatives living in residential long-term care settings. Psychology and Aging. 2024;39(5):565-577. doi:10.1037/pag0000820
10. Mittelman MS, Bartels SJ. Translating Research Into Practice: Case Study Of A Community-Based Dementia Caregiver Intervention. Health Affairs. 2014;33(4):587-595. doi:10.1377/hlthaff.2013.1334
