# Supplementary material for: The TBI-AD/ADRD Caregiver Support Intervention (TACSI): Protocol of a Pilot Randomized Controlled Trial Evaluation of a Remote Intervention for Family Caregivers
Source: JMIR Res Protoc. 2026 Mar 17;15:e81125. doi: 10.2196/81125 (PMC12994761; doi:10.2196/81125)
Supplement: Multimedia Appendix 2 [file resprot-v15-e81125-s002.docx]

Questions may be added, modified, swapped, or omitted to facilitate conversation based on the semi-structured nature of the interview. This template will be used as a general interview guide.

**Introduction**

Thank you so much for participating in our evaluation of the TACSI program. To follow-up on your participation, I would like to ask some questions about your experience.

To aid in data analysis, is it ok to audio-record our interview today? It’s ok if you prefer not to be recorded.

● If no, is it ok to take notes regarding your responses?

My objective in this interview is to determine whether and how the TACSI program benefited or did not benefit you and your <relative>/person you care for.

**Opening question**

1. Just to get us started, can you tell me a little bit more about your relative.

a. When did their TBI occur? Did they have more than one? (If yes,) When did they occur?

b. When did you first notice their dementia/memory problems?

c. When did you first start providing help to your relative because of her/his dementia or TBI?

d. What type of help or care do you provide to your relative?

e. How are things going now for you and your relative?

**Enrollment reason(s)**

2. Why did you decide to enroll in this project?

**Overall impression**

3. What was your overall impression of the TACSI program?

**Harm and drawbacks**

4. What negative outcomes, if any, resulted from being involved in the TACSI program for you?

a. Why (or how) do you think that/these happened?

5. What negative outcomes, if any, resulted from the TACSI program for your relative?

a. Why (or how) do you think that/these happened?

**Benefits**

6. What benefits, if any, resulted from involvement in the TACSI program for you?

a. Why (or how) do you think that/these happened?

7. What benefits, if any, resulted from involvement in the TACSI program for your relative?

a. Why (or how) do you think that/these happened?

8. Do you feel like your use of TACSI had any effect on your:

a. interactions with your relative?

b. interactions with other family members besides your relative?

c. interactions with your relative’s healthcare team or facility staff (if applicable)?

**TACSI components**

9. I’d like to walk through the various service components of the TACSI program and ask how each component did or did not help you or your relative and why:

a. Which individual coaching sessions or topics helped most? Least? Why?

b. Did you have any family members participate in a session along with you?

<if yes> What happened in the family sessions that helped the most? And the least? Why?

c. How did discussing your relative’s care with <coach> help or not help? <i.e. make adjustments to relative’s care, have subsequent discussions with healthcare team or family members>

d. What parts of TACSI do you think should be changed, added, or removed? Why?

**Links to outcomes**

10. Do you feel like your involvement in TACSI had any effect on your feelings of stress and burden?

11. Do you feel like your involvement in the TACSI program had any effect on your ability to care for and engage with your relative?

12. Do you feel like your involvement in the TACSI program had any effect on your mood?

**VA participant only questions:**

13a. Have you participated in any VA caregiver support programs (like the REACH VA, the one-on-one coaching program, Peer Mentor program, or Annie App)?

13b. Do you participate in the VA caregiving stipend program (i.e. PCAFC or family comprehensive)?

13c. If yes to a or b: How does the TACSI program compare to those other VA caregiver support programs?

**Ending questions**

14. Thank you for helping us learn more about your experiences with the TACSI program. Is there anything else you’d like to share with me today?
